# The “wireless” portion of a wire-reinforced endotracheal tube may kink

**DOI:** 10.1186/s40981-019-0238-x

**Published:** 2019-03-16

**Authors:** Takuhiko Wakamatsu, Hisanari Ishii

**Affiliations:** Department of Anesthesia, Tenri Hospital, Mishima-cho 200, Tenri, Nara 632-8552 Japan

**To the Editor,**

Wire-reinforced endotracheal tubes (WRETs) have a layered structure in which metal wire is embedded in the wall of the tube shaft. This structure makes the tube resistant to kinking caused by angulated forces and improves patient safety [1]. WRETs are useful for head and neck surgery during which kinking of the tube is likely to occur [2]. However, the use of a WRET may be associated with several problems causing the partial or total occlusion of the tube. There are numerous reports of obstruction of WRETs due to dissection of the layered structure [3, 4], patient bite [2, 5], or compression by surgical devices [1]. We herein report a very rare case in which a WRET became kinked and obstructed at its “wireless” portion.

A 74-year-old, 148-cm, 57-kg female patient underwent neck lymph node resection under general anesthesia. A WRET of 7.0 mm in internal diameter (Parker Flex-Tip PFRC tracheal tube, Parker medical, CO, USA) was placed orally. Volume-controlled mechanical ventilation was performed with a tidal volume of 450 ml and a respiratory rate of 10 breaths per minute. The neck of the patient was extended, and the respiratory circuit was fixed with a flexible circuit holder (ACOMA, Japan). After covering the patient’s head with surgical drapes, the surgical procedure was started. During the procedure, the surgeon moved the circuit holder to improve his working space. Subsequently, the peak airway pressure gradually increased from 16 cmH_2_O to a maximum of 28 cmH_2_O without a marked change in the waveform of capnography, increase in end-tidal CO_2_, or decrease in SpO_2_. The tidal volume setting was reduced from 450 to 390 ml, which resulted in a decrease in the peak airway pressure to 18 cmH_2_O. The surgical procedure was not aborted and was finished uneventfully. After the removal of the surgical drapes, kinking of the tube causing obstruction was observed (Fig. 1). The patient recovered from general anesthesia without complications.

**Fig. 1:**
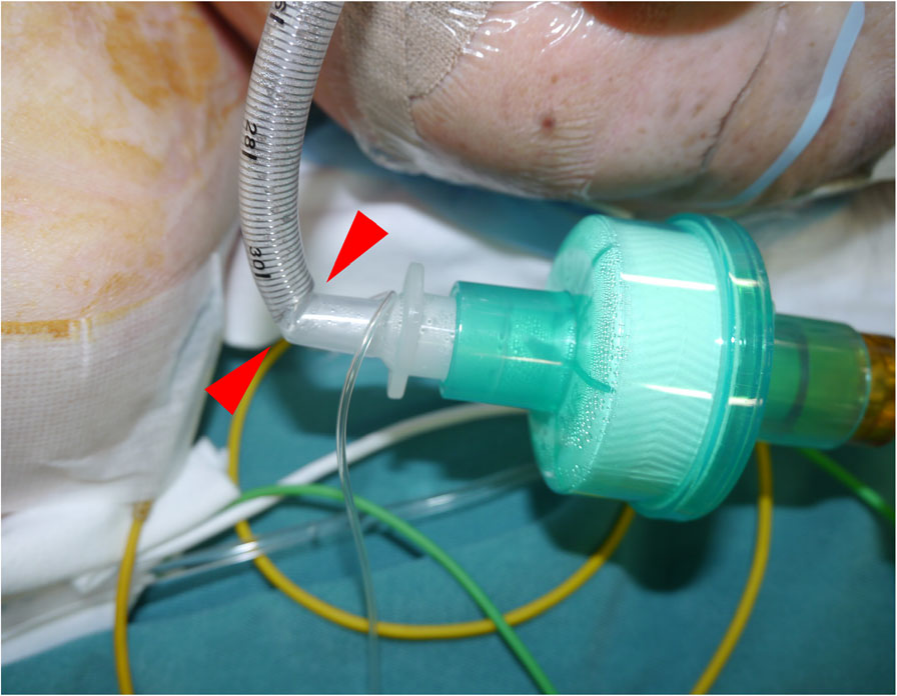
Kinking of the WRET after surgery. The WRET kinked at the portion between the end of the embedded coiled wire in the tube and the tip of the plastic slip joint (red arrowheads)

In certain types of WRETs, there is a “wireless” portion of a few millimeters between the end of the embedded coiled wire in the tube and the tip of the plastic slip joint (Fig. 2a). Kinking occurs when an angulated force is applied at this “wireless” portion (Fig. 2b). We measured the length of the “wireless” portion in Parker Flex-Tip PFRC tracheal tubes of 7.0 mm in internal diameter. We chose 10 tubes randomly from the stock in our hospital. The length of the “wireless” portion ranged from 0.4 to 2.1 mm. In our case, the length of the “wireless” portion was 1.7 mm, which was within the range for this type of tube. Moreover, the tube kinked with an angulated force that was not applied directly to the tube. Thus, kinking of WRETs may occur in clinical situations.

**Fig. 2:**
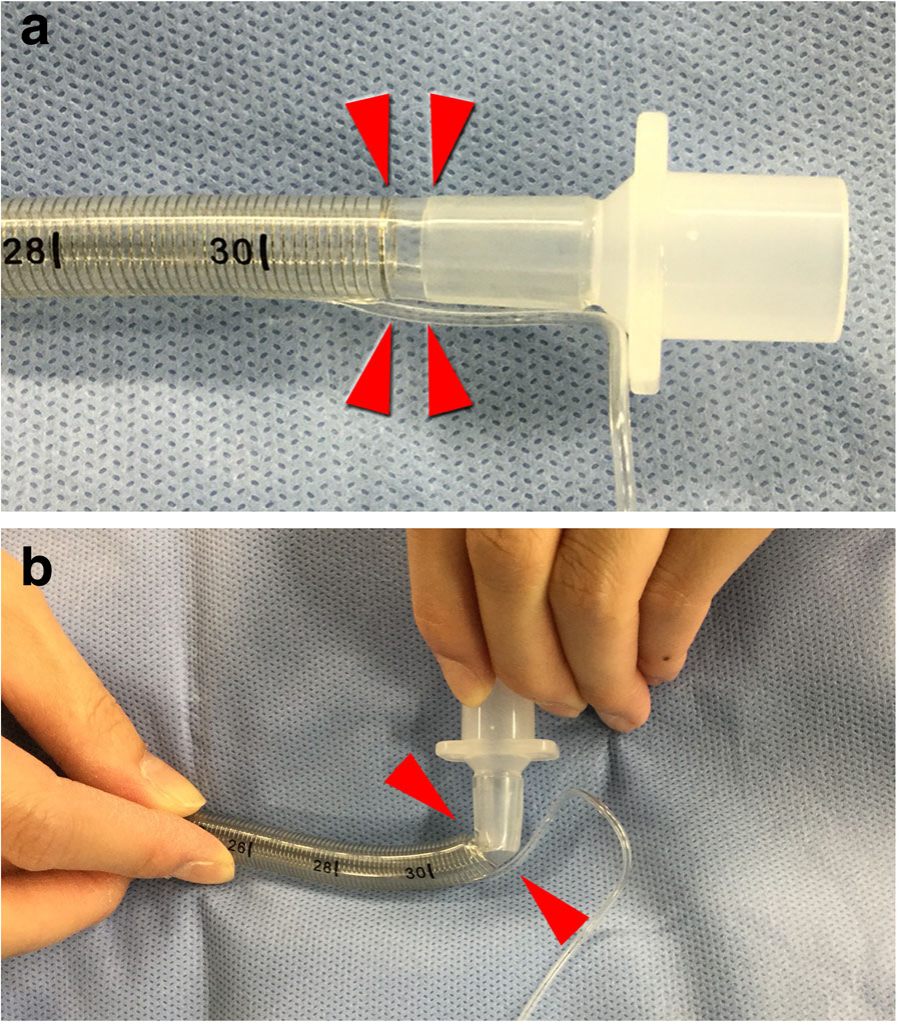
The “wireless” portion of a WRET. **a** The wireless portion between the end of the embedded coiled wire in the tube and the tip of the plastic slip joint. Red arrowheads indicate the wireless portion. **b** A WRET becomes kinked when an angulated force is applied at this portion (red arrowheads)

Certain types of WRETs have a “wireless” portion that potentially causes kinking of the tube. The endotracheal tube must be kept at an adequate angle to avoid kinking, even when using a wire-reinforced tube.

## Abbreviation

WRET: Wire-reinforced endotracheal tube
